# Predictors of Neurocognitive Syndromes in Combat Veterans

**DOI:** 10.7759/cureus.293

**Published:** 2015-07-30

**Authors:** Michael J Roy, Michelle Costanzo, Jessica Gill, Suzanne Leaman, Wendy Law, Rochelle Ndiongue, Patricia Taylor, Hyung-Suk Kim, Gayle S Bieler, Nikhil Garge, Paul E Rapp, David Keyser, Dominic Nathan, Michael Xydakis, Dzung Pham, Eric Wassermann

**Affiliations:** 1 Department of Medicine, Uniformed Services University of the Health Sciences; 2 National Institute of Nursing Research, National Institutes of Health; 3 Traumatic Brain Injury Service, Walter Reed National Military Medical Center; 4 National Intrepid Center of Excellence, Walter Reed National Military Medical Center; 5 National Institute of Nursing Research , National Institutes of Health; 6 RTI International; 7 Traumatic Injury Research Program, Uniformed Services University of the Health Sciences; 8 Traumatic Brain Injury Service, Uniformed Services University of the Health Sciences; 9 Department of Surgery , Uniformed Services University of the Health Sciences; 10 Image Processing Core, Center for Neuroscience and Regenerative Medicine, Henry Jackson Foundation; 11 Behavioral Neurology Unit, National Institute of Neurological Disorders and Stroke, National Institutes of Health

**Keywords:** traumatic brain injury, depression, posttraumatic stress disorder, combat military veterans, neuroimaging, biomarkers

## Abstract

Traumatic brain injury, depression and posttraumatic stress disorder (PTSD) are neurocognitive syndromes often associated with impairment of physical and mental health, as well as functional status. These syndromes are also frequent in military service members (SMs) after combat, although their presentation is often delayed until months after their return. The objective of this prospective cohort study was the identification of independent predictors of neurocognitive syndromes upon return from deployment could facilitate early intervention to prevent disability. We completed a comprehensive baseline assessment, followed by serial evaluations at three, six, and 12 months, to assess for new-onset PTSD, depression, or postconcussive syndrome (PCS) in order to identify baseline factors most strongly associated with subsequent neurocognitive syndromes. On serial follow-up, seven participants developed at least one neurocognitive syndrome: five with PTSD, one with depression and PTSD, and one with PCS. On univariate analysis, 60 items were associated with syndrome development at p < 0.15. Decision trees and ensemble tree multivariate models yielded four common independent predictors of PTSD: right superior longitudinal fasciculus tract volume on MRI; resting state connectivity between the right amygdala and left superior temporal gyrus (BA41/42) on functional MRI; and single nucleotide polymorphisms in the genes coding for myelin basic protein as well as brain-derived neurotrophic factor. Our findings require follow-up studies with greater sample size and suggest that neuroimaging and molecular biomarkers may help distinguish those at high risk for post-deployment neurocognitive syndromes.

## Introduction

Traumatic brain injury (TBI) and posttraumatic stress disorder (PTSD) have been called signature injuries of the Iraq and Afghanistan wars [1-2]. For a variety of reasons, service members (SMs) frequently underreport PTSD symptoms upon their initial return from combat. However, the rate of PTSD can increase as much as three-fold in the ensuing months [3], and delayed diagnosis can make treatment more difficult, leading to lower rates of return to duty, greater healthcare utilization and functional impairment, and profound loss of Quality Adjusted Life Years [4-7]. Depression can develop either independently or as a complication of PTSD and poses similar problems, as does TBI. It has been estimated that hundreds of thousands of U.S. SMs have experienced TBI [8]. In addition to direct brain injury from blunt or penetrating trauma, blast, or acceleration-deceleration forces can cause cerebral edema and diffuse axonal injury with consequent impairment in attention, memory, executive function [9], behavioral control, physical function [10-11], quality of life [12], return to work, and social activities [13]. TBI is a point of injury diagnosis, whereas consequent post-concussive syndrome (PCS) parallels the often prolonged course of PTSD. There is considerable overlap between the criteria for PTSD, PCS, and depression, favoring their study in tandem. Identifying independent predictors of these syndromes on return from deployment could facilitate early intervention to prevent disability.  

We therefore conducted a prospective cohort study involving recently deployed active duty and reserve component U.S. military SMs who did not meet criteria for PTSD, depression, or PCS at the time of enrollment. Within two months after the SMs returned from deployment, we completed a comprehensive baseline assessment, including demographics, analysis for single nucleotide polymorphisms (SNPs) in neuropeptide and stress regulation genes, neuroendocrine assays, brain imaging and electroencephalograms, vestibular, olfactory, and psychophysiologic measures. The baseline measures were chosen because of their potential to influence outcome after a deployment, either with or without a TBI. For example, single-nucleotide polymorphisms (SNPs) have been linked with PTSD in several studies [14-16]. Follow-up evaluations at three, six, and 12 months were then conducted to assess for development of PTSD, depression, or PCS. The intent was to develop a risk stratification model incorporating independent predictors of clinically significant post-deployment neurocognitive syndromes.

## Materials and methods

### Participants

Participation required deployment for at least three months to Iraq or Afghanistan, and travel to Bethesda, MD for a baseline assessment within two months after their return. Exclusion criteria were: an unstable medical condition; active suicidal or homicidal ideation; psychosis; loss of consciousness for more than 60 minutes; a diagnosis of PCS according to criteria from the International Classification of Diseases, 10th Clinical Modification (ICD-10); a Patient Health Questionnaire-9 (PHQ-9) score > 10 [17]; and a PTSD Checklist-Military version (PCL-M) score > 50 [18], or a diagnosis of PTSD made by an experienced psychologist on the Clinician Administered PTSD Scale (CAPS) [19]. The study was open to all active duty military service members who have served in Iraq or Afghanistan, regardless of rank, race, age, or gender. Participants volunteered after seeing information about the study on the Center for Neuroscience and Regenerative Medicine (CNRM) website, Facebook page, or after contact with CNRM recruiters during immediate post-deployment demobilization procedures at Fort Dix, NJ. Eighty-five individuals gave written informed consent and completed the baseline assessment between December 2011 and June 2012. Two individuals were excluded for PCL-M scores > 50, and two for PHQ-9 scores > 10. Among the resulting cohort of 81 participants (11 women), 69 completed at least one follow-up assessment, including 54 at three months, 39 at six months, and 57 at 12 months. The mean age was 29.7 at baseline (SD 7.9: range = 19 – 51); 9 identified themselves as African-American, 53 White, 5 Asian, 6 Hispanic, and 8 were unknown. Thirty-six were enlisted, 17 were officers, and 28 declined to state their rank. Participants reported a mean of 9.5 years of service (SD 5.6) and a mean of 1.7 deployments (SD.96). Their current duty stations spanned the country, representing the following states and territories: AL (1), AZ (1), CA (5), CT (1), FL (6), IA(3), IL (3), KY (1), LA (1), MD (8), MN (5), NC (5), NE (2), NV (12), NY (2), OH (2), PA (8), PR (1), SD (4), TN (4), VA (4), WA (1), and WV (1). The study was approved by the Institutional Review Boards from Walter Reed National Military Medical Center, Uniformed Services University, and National Institute of Neurological Disorders and Stroke.

### Baseline evaluation

The principal investigator, a board-certified internist, obtained written informed consent from each participant and then performed a medical history and physical examination; the latter included the Romberg, finger-to-nose, heel-to-shin, and rapid alternating movement tests, and assessment of gait, pronator drift, and eye movements. Olfactory function was assessed with the University of Pennsylvania Smell Identification Test (UPSIT) [20]. Vestibular and olfactory function have frequently been impaired after moderate to severe TBI but have not been carefully studied in SMs with mild or no TBI. The Defense Veteran’s Brain Injury Center (DVBIC) screen was used as an initial screen for a history of TBI [21]. All participants also completed the CAPS, PCL-M, and full Patient Health Questionnaire and were evaluated for ICD-10 criteria for PCS.

Blood samples were collected from all study participants at rest to assess for SNPs in genes that may to be related to the risk of developing neurocognitive syndromes, including neuron-specific enolase, myelin basic protein (MBP), brain-derived neurotrophic factor (BDNF), and S100B, as well as plasma catecholamines and serum cortisol. We assessed psychophysiological responses with two paradigms: First, we administered a validated fear acquisition and fear extinction experiment [22-24] and then assessed physiological responses (heart rate, blood pressure, respiratory rate, electromyography eye-blink startle, skin conductance level) to three 2-minute sequences in a highly realistic virtual Iraq/Afghanistan environment [25-26]. Catecholamine measurements were repeated immediately after both paradigms. Event-related potentials were recorded at sites Fz, Cz, Pz, Oz, C3, and C4 during an oddball task. In addition to standard structural brain magnetic resonance imaging (MRI), resting state blood oxygen level-dependent functional MRI (fMRI) [27-29] and diffusion tensor imaging (DTI) [30-34] were performed. Each of the measures was chosen because we and colleagues have also employed them in other studies in which they have had value, though their utility in predicting subsequent neurocognitive syndromes has not been previously assessed. The psychophysiological and imaging methods are further characterized below.

Psychophysiology

Visual stimuli were presented through SuperLab 4.0 for Windows, and acoustic stimuli were presented with noise canceling stereo earphones. The aversive stimulus (also known as the unconditioned stimulus, US) was a 250 ms airblast with an intensity of 140 p.s.i. directed to the larynx as has been previously described in similar human fear conditioning studies [22-24, 35-36]. Airblasts were emitted by a compressed air tank connected to polyethylene tubing and controlled by a solenoid switch. Conditioned stimuli (CS’s) were colored shapes presented on a computer monitor. The colored shapes were counterbalanced across subjects. Note: the use of geometric shapes allows the investigators to include participants that may have color blindness as they can still be easily ascertained as different stimuli. The task began with a Habituation Phase consisting of six acoustic startle probes presented alone (noise alone (NA) trials) to reduce initial startle reactivity, and followed by a stimulus Pre-exposure Phase during which the subject saw the shapes (A, B, and X) but they were not paired with the US. The Acquisition Phase includes three blocks with 12 trials (4 AX, 4 BX, and 4 NA trials) in each block for a total of 36 trials. The Inhibition Testing Phase consisted of a block of three NA trials and three trials with A and B presented together; the Fear Extinction Phase was presented with six blocks of 12 trials each (4 AX, 4 BX, and 4 NA trials). In the AX+ trials, two shapes of different colors were presented together with a “+” between them to encourage elemental processing of the shapes. The shapes were presented for 6 seconds. The startle probe was then presented after 6 seconds and was followed by the US 500 msec later. BX and AB trials also contained two different colored shapes, but there was no US in these trials. During the Fear Extinction Phase, none of the AX or BX trials was reinforced with the airblast US. Intertrial intervals were randomized between 9 and 22 seconds.

For the VR sequence, video clips were presented though SuperLab 4.0 for Windows. The session was initiated with a 30-sec presentation of a blue square during which time two 108-dB, 40-ms startle probes were presented. The time between the startle probes was randomized between 9 and 22 sec during the blue screen. A two-minute VR-type video was played depicting a soldier's position at the gunner position on the roof of a Humvee. Combat-related stimuli, including smoke, gunfire, explosions, and roadside insurgents, were presented during the two-minute clip in an ascending order of severity. Six startle probes were presented throughout the video clip at time points in which combat-related audio stimuli were minimal. A 30-sec blue screen was again presented with two more startle probes 9-22 sec apart. A second two-minute VR-type video was played depicting a soldier's position within the cabin of a Humvee. Again, combat-related stimuli, including smoke, gunfire, explosions, and roadside insurgents, were presented during the two-minute clip in an ascending order of severity. Five startle probes were presented throughout the video clip at time points in which combat-related audio stimuli were minimal. A 30-sec blue screen was again presented with two more startle probes 9-22 sec apart. A third two-minute VR-type video was played depicting a soldier's position as he/she walks through the streets of Baghdad. Once more, combat-related stimuli, including smoke, gunfire, explosions, and overhead aircraft, were presented during the two-minute clip in an ascending order of severity. Four startle probes were presented throughout the video clip at time points in which combat-related audio stimuli were minimal.

Psychophysiological data was recorded with Biopac MP150 for Windows (Biopac Systems, Inc., Aero Camino, CA) sampled at 1000 Hz, digitized at 16 bit A/D resolution, and amplified. Using the Biopac Acknowledge software, EMG was Band Pass 28-500Hz and rectified [37]. Difference scores were computed from the peak amplitude recorded between 20 and 200 ms after the startle probe offset [37]; thus each startle probe was based on the [startle magnitude in the presence of a CS in each conditioning block] - [startle magnitude to the noise alone] [22]. GSR was 1 Hz low-pass filtered and a 0.05 Hz high-pass filtered. GSR response score for each CS was calculated by subtracting the mean level for the 2 sec immediately preceding CS onset from the highest value among those recorded during the 6 sec CS interval [38]. ECG was Band Pass filtered from 0.5-35Hz. ECG was converted to heart rate in beats per minute. HR response score for each CS was calculated by subtracting the mean HR for the 2 sec immediately preceding CS onset from the mean HR recorded during the 6 sec CS interval [38]. EMG was band-pass filtered from 28-500Hz and rectified [37].

During the VR sessions, the EMG peak amplitude 20-200 ms after the startle probe offset [37] was used to compute difference scores for each startle probe (EMG startle) by using the startle magnitude in the presence of VR, minus the startle magnitude to the startle probe delivered during the ITI [23]. During each VR sequence, mean HR, RR, and SC were computed during the two-minute viewing period consistent with previous work utilizing a multivariate approach to examining the predictive value of psychophysiology and mental state [39]. EMG startle was averaged during each VR sequence.

Neuroendocrine Measures and Biomarkers

Blood samples were drawn between the hour of 0800 and 0900 at the baseline and at the follow-up visits (three, six, and 12 months) to identify single nucleotide polymorphisms (SNPs) in: COMT, 5HTT, DAT, NSE, S100B, and MBP. Assessment of biochemical levels of IL-6, IL-10, Heat shock protein (HSP) 60, 70, and 90, and S100A12, and neuroendocrine measures (e.g., the catecholamines epinephrine, norepinephrine and dopamine, as well as cortisol were completed. SNPs within genes of interest were genotyped using the 5’ nuclease allelic discrimination assay (TaqMan Assay). Thermal cycling and end-point PCR were performed on an ABI PRISM 7900HT Sequence Detection System. TaqMan assays are based on dual-labeled fluorogenic probes for each allele of each SNP. Real-time polymerase chain reaction (PCR) via this method measures the accumulation of fluorophore and thus determines the genotype based on the amount of product for each allele. Protein concentrations were determined by a commercially available sandwich enzyme-linked immunosorbent assay (ELISA). Patient plasma and/or serum samples were prepared according to manufacturer recommendations. All ELISA microplates were read by a SPECTRAmax Ms ROM v2.00b73 (Molecular Devices, Sunnyvale, CA). Known standards were included on all plates, and unknown samples were assayed in duplicate. Concentration values with coefficients of variation (CV) > 10% were re-assayed. The protein concentrations were determined, using ELISA methods as described above, for IL-6, IL-10, HPA axis measures (cortisol and adrenocorticotropin hormone (ACTH), and modulators of the response to chronic and acute stressors, including neuropeptides (neuropeptide –Y and galanin) and neurotrophins (brain-derived neurotrophic factor and insulin-like growth factor).

Genome-wide association study (GWAS) was conducted with the Affymetrix Gene Chip Assay SNP 6.0 (Affymetrix, Santa Clara, CA), by scanning for 500 nanograms of genomic DNA from peripheral blood following manufacturer instructions. Briefly, genomic DNA was digested, ligated, and amplified using PCR. The PCR product was cleaned, fragmented, labeled, and hybridized onto the array and incubated for 17 hours in the hybridization chamber. The hybridized array was scanned using the Affymetrix Scanner 3000-7G with autoloader (Affymetrix, Santa Clara, CA). All samples passed the quality control (Contrast QC greater than 0.4).

EEG and ERP Oddball Task

Supraliminal visual stimuli were presented by a digital tachistoscope of our own design and construction. The tachistoscope was a square 5 x 5 array of yellow, light-emitting diodes. Each diode was 1 cm in diameter. Two LED stimulus patterns were used. The vertical stimulus consists of the five central LEDs illuminated simultaneously for 40 msec. The second stimulus, the horizontal stimulus, was composed of the five horizontal center line LEDs illuminated simultaneously for 40 msec. The inter-stimulus onset time was varied randomly between 1.4 and 1.8 seconds. The experimental protocol for EEG recording:

- Free running EEG, eyes closed, no task, 2.5 minutes

- Free running EEG, eyes open, no task, 2.5 minutes

- Horizontal stimulus (Target) 25 trials, Vertical stimulus, 100 trials, approximately 4 minutes

- Horizontal stimulus 75 trials, Vertical stimulus (Target) 50 trials, approximately 4 minutes

- Horizontal stimulus (Target) 50 trials, Vertical stimulus 75 trials, approximately 4 minutes

- Horizontal stimulus 100 trials, Vertical stimulus (Target) 25 trials, approximately 4 minutes

- Free running EEG, eyes open, no task, 2.5 minutes

- Free running EEG, eyes closed, no task, 2.5 minutes

There was a brief break between each component. During tachistoscope presentations, the participant was instructed to maintain a silent count of the number of target stimulus presentations. They were told that they can neglect appearances of the standard stimulus. The participants were given accuracy feedback after each report. In order to not cue the participant, the target/standard presentation ratios were varied slightly from the values given above. For example, instead of 75/50, a 78/47 ratio might be used.

The recording methods were noninvasive and the visual stimuli that were presented were nonaversive, emotionally neutral horizontal or vertical lines. The EEG experiment was not physically demanding and participants were seated comfortably throughout the experiment. The EEGs were read by a physician holding subspecialty qualifications in electroencephalography. The EEG equipment that was used has prior US Food and Drug Administration (FDA) approval for use with human participants. All recording procedures followed normal longstanding electroencephalographic practice.

EEG and ERP Analysis Methods

A single trial was defined as the epoch incorporating the pre-stimulus baseline extending to an upper bound after stimulus onset. Correction of blink artifact was performed by removing the electrooculogram (EOG) signal from each channel. This was achieved through the use of a ‘least squares’ regression function to estimate the amount of EOG signal that was present in a specific EEG channel [40-41]. Intermittent bursts of high-frequency electrical noise were eliminated from the analysis; the corrupted trial was eliminated for all electrode sites (even if a burst was confined to one channel or a limited number of channels). In the oddball task, the N_Target_ was the number of qualified target single trials, the N_Standard_ was the number of qualified standard responses, and the average voltage signal V_Target_ (t) was the time series formed by averaging qualified single-trial responses to the target stimulus. The peak amplitude of the P300, the latency of the P300, and the area under the curve of the P300 wave were calculated. Special MatLab programs were designed for each and are available upon request. A zero-phase, fifth-order low-pass Butterworth filter with a cut-off frequency of 5Hz was used to obtain the envelope of the average ERP epoch (-200 ms to 1000 ms). From this ERP envelope, the peak amplitude was measured as the maximum value of the signal. The latency measure was calculated as the time from the target to the peak amplitude. The area under the curve measure was obtained by calculating the integral of the absolute area under the curve of the epoch envelope for the entirety of its duration (-200 ms to 1000 ms).

Vestibular and Olfactory Function

A clinical assessment of vestibular function by the principal investigator included examination of extrinsic ocular muscles, assessment for nystagmus by checking lateral gaze to each side, and assessment of cerebellar function to include the Romberg test, pronator drift, finger-to-nose, heel-to-shin, and rapid alternating movement testing. Tandem gait, heel-to-toe walking in a straight line, and walking on toes and heels was also assessed. If any abnormalities were detected on clinical vestibular assessment, the plan was to perform videonystagmography, rotary chair testing, air caloric testing, vestibular evoked myogenic potentials, unilateral centrifugation testing, vertical axis rotation, and subjective visual vertical and horizontal testing. However, no study participants had abnormal clinical assessments, so more detailed testing was not performed.

To test olfactory function, the University of Pennsylvania Smell Identification Test (UPSIT) [Semsonics, Inc, Haddon Heights, NJ] was used since it is considered the gold standard in the United States. The UPSIT focuses on the comparative abilities of individuals to identify a number of odorants at the supra-threshold level. It is a standardized 40 stimulus microencapsulated “scratch and sniff” test. The UPSIT test categorizes individuals into five distinct levels of olfactory functioning: normosmic (normal), mildly, moderately, and severely hyposmic (impaired functioning), and anosmic (no sensation). The UPSIT is a four alternative, forced-choice microencapsulated odorant test. Physically, the UPSIT is comprised of four test booklets, each containing 10 pages. A strip embedded with a microencapsulated odorant was present on the bottom of each page, just below a four alternative multiple-choice question. For a given item, the patient releases an odor by scratching the microencapsulated label with a pencil tip, smells the label, and indicates the odor quality from four alternatives. Even if no smell was perceived, a response was required. The subject’s total correct score out of the 40 items was determined. This score was then compared to scores in a normative database, providing an indication of the level of absolute smell function and a percentile rank for each age and gender group. The subjects were tested individually in a quiet room. To acquaint the patient with the testing procedure, a practice trial was given to verify adequate comprehension of the task. Responses were read aloud by the examiner for accuracy with questioning. The odorant was presented approximately 2 cm in front of both nostrils for 2 seconds. Both nostrils were tested simultaneously. The assessment consisted of a successive presentation of each of 40 familiar micro-fragrance odorants in a single trial. After announcing the presentation of the next stimulus, the examiner scratched the label twice while holding it immediately below both nostrils of the patient. The subject could use any convenient sniffing strategy but should sniff in a consistent fashion from trial to trial.

*Resting-state fMRI*

Resting-state fMRI images were acquired on a Siemens Biograph mMR 3T scanner with a gradient echo echoplanar imaging (EPI) sequence (TR=2000 ms, TE=27 ms, Flip angle=90 degrees, Voxel size = 3.43x3.43x3 mm with 0.6 mm gap, Matrix size=64x64, Slices=36, Timepoints=206). Images were processed using the DPARSF/REST Toolkits [27,28], which are based on the Statistical Parametric Mapping (SPM 8) software (*http://www.fil.ion.ucl.ac.uk/spm*). The first 10 volumes were discarded and the remaining images were corrected for motion and slice timing. Images were then co-registered to T1-weighted MPRAGE (Magnetization Prepared RApid Gradient Echo) images (TR=2530 ms, TE=3.03 ms, TI=1100 ms, Flip angle=7 degrees, Voxel size=1x1x1 mm, Matrix size=256x256, Slices=176). Normalization into Montreal Neurological Institute (MNI) space was performed using the diffeomorphic anatomical registration through exponentiated lie algebra (DARTEL) algorithm in SPM. The final resolution of all images was 2x2x2 mm. Spatial smoothing was then applied at 8 mm full-width half-max, followed by temporal bandpass filtering (0.01-0.12 Hz). Nuisance effects due to motion, cerebrospinal fluid blood-oxygen-level-dependent (BOLD) signal, and white matter BOLD signal were regressed from the images and residuals were used for subsequent analysis. For functional connectivity analysis, regions of interest (ROIs) were defined by deformable registration of the Harvard-Oxford structural atlas [29], resulting in a total of 112 cortical and subcortical regions. Pearson correlation coefficients were computed between all ROIs and converted into z-scores using Fisher’s transformation.

Diffusion Imaging

Diffusion-weighted images were acquired on a Siemens Biograph mMR 3T scanner with parameters TR=17000 ms, TE=98 ms, Flip angle=90 degrees, Voxel size=2x2x2 mm, Matrix size=128x128, and Slices=75. The acquisition included 10 images at b=0 s/mm^2^, 10 images with non-collinear directional gradients at b=300 s/mm^2^, and 60 images with non-collinear directional gradients at b=1100 s/mm^2^. Images were processed using the CATNAP software described in [30] for tensor estimation. Briefly, images were preprocessed for motion correction and eddy current correction, with adjustments to the gradient table performed based on patient position. Distortions due to echo planar imaging susceptibility artifacts were corrected by performing a deformable registration to an anatomic T2-weighted acquisition (TR=3200 ms, TE=409 ms, Flip angle=120 degrees, Voxel size=1x1x1 mm, Matrix size=256x256, Slices=176). Linear tensor estimation was performed followed by computation of fractional anisotropy (FA), segmentation of white matter tracts with the DOTS software [31], and fiber tracking with TrackVis [32]. Fiber tracking for the cingulum bundle was performed similarly to the approach described in [33]. Regions of interest in the anterior cingulate gyrus and posterior cingulate gyrus were determined automatically by atlas registration of the Harvard-Oxford structural atlas to a co-registered T1-weighted image using the ANTS software [34]. The cingulum bundle was then defined using FACT fiber tracking in TrackVis to pass through both regions of interest. The maximum angle threshold was set to 35 degrees and the FA threshold was set to 0.15. Additional regions of interest were manually placed to prevent fiber tracking from connecting into adjacent bundles.

### Three month, six month and 12-month follow-up

At the follow-up visits, we used the CAPS, PHQ-9, and ICD-10 criteria for PCS, to determine the presence of PTSD, depression, and PCS, respectively.

### Statistical analyses

All analyses were performed with the R statistical software package (R Development Core Team, 2011). Univariate analysis was used to identify baseline predictors significant at *p* < 0.15, to allow a more inclusion group of independent variables to be included in a multiple regression. This involved performing logistic regression (since the outcome was binary) on each of the baseline measures individually and independently. Continuous variables were modeled with a single regression coefficient. A positive regression coefficient for any predictor suggests an increase in the probability of PTSD with increases in the predictor; a negative regression coefficient suggests a reduction in the probability of PTSD with increases in the predictor. Categorical predictors (demographics such as ethnicity) were modeled using the reference cell parameterization approach:  that is, we included the K-1 indicator variables corresponding to a given categorical predictor simultaneously in the model, where K is the number of levels for the predictor, and each indicator is a 0/1 variable denoting membership in that level.

Since some measures, such as imaging patterns or some of the genetic factors, might be closely related to each other (multicollinearity), a variance inflation factor (VIF) method [42] was used and baseline measures with VIF > 10 were excluded. A predictive modeling approach utilizing stepwise multivariate decision trees (also known as a Classification and Regression Tree, or CART approach) [43] was employed on the significant baseline variables obtained through univariate and multicollinearity analyses. However, single-tree models can be sensitive to small changes in the data. A slight change in a data set can result in a different tree structure, thereby inducing high variability in predictions obtained across trees. Therefore, ensemble methods, such as random forests [44], are commonly exercised to build a large number of tree models (on bootstrap samples) and aggregate predictions across trees to obtain stable predictions. Generally, a positive variable importance (VIMP) indicates that the variable is associated with the outcome while VIMP <= 0 indicates no association. Thus, the random forests approach was also used to rank the baseline measures in their order of importance in predicting 12-month follow-up of PTSD using 1000 trees on the same variables obtained through univariate and multicollinearity analyses. Both the CART and random forests approach are machine learning methods that are specifically designed for situations where the number of potential predictors may exceed the number of observations. Given that classical statistical approaches of logistic regression and linear discriminant analysis for classification problems break down when the data are highly dimensional, CART and random forests were applied since these machine learning approaches are designed for situations where the number of potential predictors is far greater than the number of observations. The CART individual trees technique automatically sifts large, complex databases, searching for and isolating significant patterns and relationships, which help to generate predictive models [43]. Random forests represent a supervised learning approach to a known outcome that we are trying to predict—in this case, the development of neurocognitive syndromes—and generally has superior performance [45-46].

## Results

Vestibular and olfactory assessments were uniformly normal and, therefore, excluded from further analyses. We then compared 555 baseline measures as independent variables versus the primary outcome of interest as a dependent variable: the development of PTSD, PCS, or depression over the course of 12 months. Sixty-nine subjects completed at least one follow-up visit and were included in this analysis, divided between seven cases (one PCS, one PTSD and depression, and five PTSD) and 62 controls. Eleven had a history of combat mTBI, all with no more than transient loss of consciousness. The univariate analysis identified 60 baseline measures, which were associated with the outcome at a *p* < 0.15 level, and these variables were then included in multivariate analyses for predicting the most prevalent syndrome that developed in this cohort, PTSD (see Table 1 in the Appendix).

**Figure 1:**
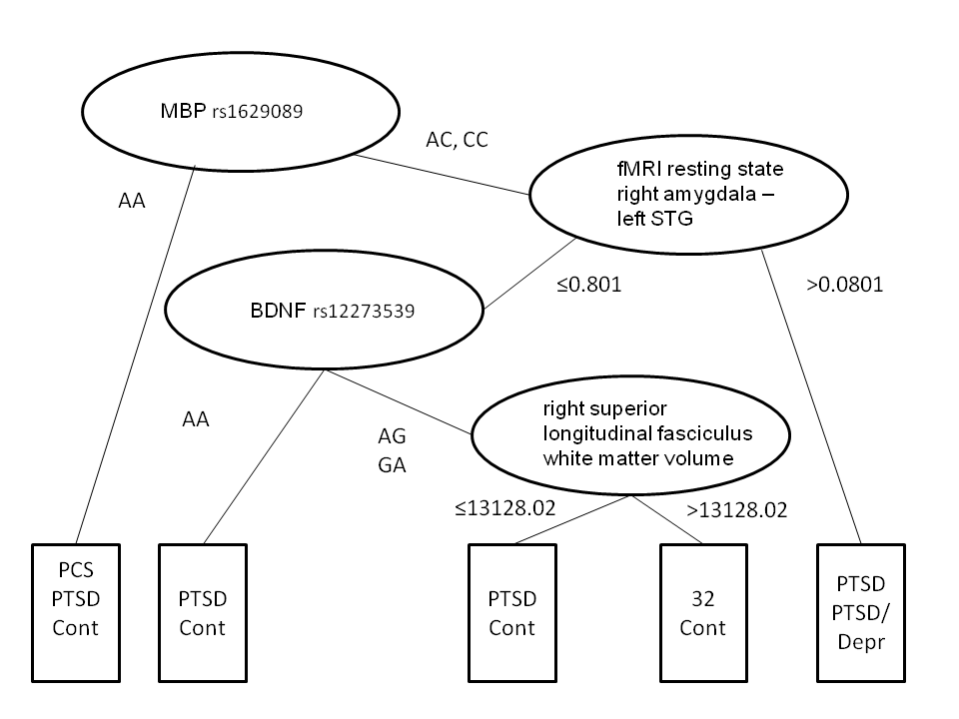
Multivariate analysis using decision tree (CART). Visual representation of final result of the decision trees.

Before performing multivariate analyses, the significant baseline measures were assessed for multicollinearity, and a total of 21 variables—all representing either fMRI or genetic markers—were excluded, since they had a VIF > 10. In addition, ethnicity was excluded since it was not accurately reported by a number of subjects (see Table 2 in the Appendix). Finally, some subjects who had one or more remaining significant variables missing were excluded, leaving 41 subjects (seven with a history of combat mTBI) for multivariate analyses: five SMs who developed PTSD and one SM who developed PCS during follow-up, and 35 who did not develop a neurocognitive syndrome. When all 38 remaining significant baseline measures were considered simultaneously in a multiple regression model, none emerged as significant independent predictors of PTSD. However, four baseline measures emerged as significantly associated with the development of a neurocognitive syndrome with each of two different more sophisticated multivariate techniques. The CART model classified the six cases into four terminal nodes, one of which contained only cases, and the others, a combination of controls and cases (Figure 1).

**Figure 2:**
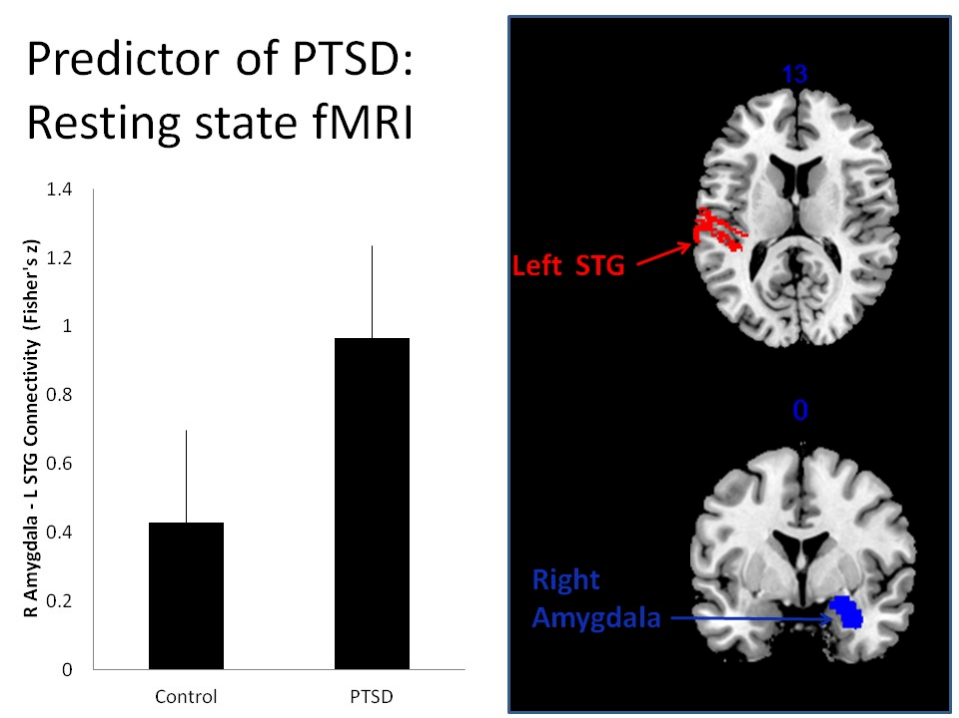
Resting state functional connectivity at baseline (2 months after deployment to Iraq or Afghanistan). Plot of controls vs. cases from the homogeneous node defined by an SNP in the gene coding for MBP (rs1629089) and resting state connectivity functional connectivity between the right (R) amygdala and left (L) superior temporal gyrus (STG). Left panel is the Pearson’s correlation (Fisher’s z ) between these regions that was significantly associated with development of PTSD (PTSD) and those who did not develop PTSD (control) during 12 month longitudinal follow-up. The right panel illustrates the anatomical locations of the left STG (axial, z-axis MNI coordinate) and right amygdala (coronal, x-axis MNI coordinate).

In addition, the overwhelming majority of the controls were contained in a single homogeneous node. The homogeneous node for cases was defined by an SNP in the gene coding for MBP (National Center for Biotechnology Information, or NCBI, database number rs1629089) and resting state connectivity between the left superior temporal gyrus (STG, BA41/42) and right amygdala > 0.801 (Figure 2), while the homogeneous node for controls was defined by the SNPs for MBP and BDNF (NCBI number rs12273539), reduced resting state connectivity between the left STG and right amygdala, and greater right superior longitudinal fasciculus (RSLF) volume.

The CART model was corroborated by the random forests approach. Of the 19 variables with VIMP scores greater than 0 (see Table 3 in the Appendix), indicating some association with outcome, the same four variables (MBP and BDNF SNPs, resting state connectivity between the right amygdala and left superior temporal gyrus (BA41/42), and RSLF volume) were among the top 10 most strongly linked measures.

## Discussion

Since PTSD and TBI-related postconcussion symptoms are often identified after deployment [47], the ability to risk-stratify service members upon return could facilitate fruitful early intervention. We report functional imaging and genetic markers, which distinguish SMs who went on to develop often-identified neurocognitive syndromes (PTSD, depression and/or PCS) in the year after completing deployment to a combat environment. The four factors that were associated with development of neurocognitive syndromes each may improve our understanding of combat-related neurocognitive syndromes.

Our identification of two SNPs in genes related to neuronal recovery, in conjunction with differences in brain structure and function, has important implications for the risk of developing neurocognitive syndromes following deployment. MBP is a major component of myelin, which is essential for axonal transmission and support. Myelin pathology may occur in PTSD, depression and PCS, and MBP is essential for remyelination after damage; SNP-related alterations in the gene which codes for this key protein could thus engender variability in recovery after mechanical or non-mechanical trauma [48]. Alterations in oligodendrocytes or myelin integrity have been linked with disruptions in connectivity between disease-relevant brain regions in stress-related disorders [49]. Increased MBP concentrations in peripheral blood have been reported after TBI [50], and greater activity is associated with better recovery from TBI [51]. Alterations in MBP have been linked to both neurologic disorders in which stress seems to play a role, such as multiple sclerosis and psychiatric disorders (such as bipolar disorder). Moreover, the rs1629089 SNP is a candidate mutation in schizophrenia [52]. We note that this SNP, in particular, distinguished the sole study participant who developed PCS during the course of the one-year follow-up, and one of the other two who manifest the SNP developed PTSD, providing further evidence of a potential role in neurocognitive disorders.

After taking into account the rs1629089, the next significant branch point in our decision tree analysis is defined by baseline resting state connectivity between the left superior temporal gyrus (STG) and right amygdala on fMRI. The left auditory cortex plays a central role in speech processing [53]; abnormal activation in this region has been reported in auditory verbal hallucinations in schizophrenia [54] with elevated resting state activity hypothesized to be pathological [55]. The STG is thought to encode echoic memory [56], so it could be postulated that elevated resting activity in the auditory cortex primes the brain for enhanced vulnerability to sensory impressions. Re-experiencing symptoms in PTSD are often tied to sensory components of the traumatic event and can be triggered by stimuli with similarities to the trauma [57]. The amygdala is integral to emotion-related behavior and plays a central role in hypervigilance (threat detection), fear conditioning and expression, and recurrent emotional memories in PTSD [58]. One aspect of interest is whether the laterality of temporal lobe involvement is significant. Our analyses identified left STG-amygdala connectivity, as did a recent study of anxious individuals under threat during a stimulus deviance task [59]; however, another recent resting state fMRI study in PTSD highlighted a role for connectivity with the right temporal lobe [60]. Nevertheless, with regard to the amygdala, the right side significance we identified is consistent with other studies of resting state fMRI in PTSD [61], and activity in this region reportedly correlates with PTSD symptom severity [62-63]. Indeed, the right amygdala seems to be more responsive to global emotional stimuli than the left amygdala, with the latter responding more to specific emotions and encoding variations in affective magnitude [64]. Further study is needed to better understand the significance of laterality in what seems to be a significant pathway for hypervigilance and hyperarousal.

In those who did not demonstrate greater resting state connectivity between right amygdala and left auditory cortex, the BDNF biomarker was the next distinguishing factor with regard to development of the neurocognitive syndromes of interest. This was not unanticipated, since BDNF is vital to learning and adaptive stress responses, influencing synaptic plasticity and connectivity, and enhancing neuronal survival [65]. Our multivariate analyses suggest a link between rs12273539 and PTSD risk, as one of two study participants who were homozygous for a guanine-adenosine SNP developed PTSD, whereas only one of the 34 subjects without this homozygous mutation did. This SNP has been previously linked to vulnerability to depression [66] and schizophrenia [67]. Our findings suggest that it should also be further studied in PTSD.

The final branch point in our CART analysis is determined by right superior longitudinal fasciculus (SLF) volume among those who were not homozygous for the rs12273539 SNP. The SLF is an association white matter fiber tract that connects the frontal lobe with the parieto-temporal regions [68]. Reduced white matter volume in this tract has been associated with TBI [69], depression severity [70], anxiety-related personality traits, and susceptibility to psychiatric disease [71]. Since our primary outcomes were incident PCS, depression, and PTSD, our findings are consistent with a growing body of literature concerning the significance of this tract. However, it should be noted that reduced volume was found in only two participants, one of whom developed PTSD, whereas it was absent in 32, none of whom developed an outcome of interest. A recent meta-analysis of white matter microstructure and PTSD identified the cingulum and the superior longitudinal fasciculus as key white matter pathways [72], and our findings support the significance of the latter.

There are some notable limitations to our study. The most significant is the relatively small size of our cohort, along with the limited fraction that developed an outcome of interest. The sample size was further diminished by those who did not return for follow-up assessments and by missing data, all of which limits the power to demonstrate associations between baseline measures and subsequent outcomes. These limitations are offset in part by the broad representation of the study population, including both active and reserve component SMs from across the US, with demographics mirroring those of the overall military population. Nevertheless, we only had one participant each with depression and PCS, respectively, and while PTSD was more common, the nodes of significance on the CART analysis each contained only one or two cases. While the small study group prevented us from developing a risk stratification model, the results do set the stage for future studies with larger numbers. We have good evidence that olfactory and vestibular testing is not useful in SMs without moderate to severe TBI. In contrast, we believe that fMRI and genetic studies may have particularly high yield but confirmation through further study is essential. Our findings should be viewed primarily as hypothesis-generating rather than definitive, and future studies should focus upon imaging, genetics, and other promising factors in a large number of individuals. For example, while they did not remain significant in our multivariate analyses, other previously published analyses looking solely at our baseline measures suggest that psychophysiological measures also warrant further study [26, 73].

## Conclusions

In summary, our prospective cohort study provides evidence that it may be possible to use measures, such as genetic markers and functional neuroimaging obtained after deployment, to identify SMs at risk for developing disabling neurocognitive syndromes in the ensuing year. The genetic factors we single out in this post-deployment population are particularly interesting, as MBP and BDNF have both been shown to increase in a dose-dependent way following TBI and are associated with recovery [74]. Moreover, key SNPs or other genetic variations in linkage disequilibrium with those SNPs, may impair this process. Recent reports of white matter protection and improved outcomes with thyroid hormone replacement therapy [75] as well as omega-3 (ω-3) fatty acids [76] following neuronal injury—the former mediated by BDNF activity and the latter through MBP—suggest the potential for genetic studies to lead to the development of preventive and therapeutic measures. Our findings suggest the possibility that MBP and BDNF work in concert to protect against or enhance recovery from brain injury, mediating the risk of long-term mechanical and psychological injury. Further work is needed to define the value of these and other predictors and, ideally, to develop a risk stratification model, which could enable targeted, timely intervention to prevent progression of symptoms.

## Appendices

**Table 1:** Univariate analysis results These results represent baseline predictors significant at *p* < 0.15. This involved performing logistic regression (since the outcome was binary) on each of the baseline measures individually and independently.

Variable	Estimate	p-value
rs9903602 biomarker	-1.757251941	0.013528753
DTI right inferior longitudinal fasciculus white matter integrity (FA)	-41.26747553	0.020162484
DTI right superior longitudinal fasciculus white matter integrity (FA)	-41.55211353	0.020933201
DTI right optic radiation white matter (FA)	-29.80132982	0.024121542
Ethnicity	2.505525937	0.026220005
rs2008323 biomarker	1.239786061	0.027344278
DTI right posterior thalamus radiation white matter integrity (FA)	-41.16834894	0.038922733
rs642506 biomarker	-1.488227812	0.042582726
ERP Average Latency Ratio -- PZ	3.937982491	0.043668331
Resting state right amygdala - left subcallosal cortex	3.979815018	0.044193087
DTI left inferior longitudinal fasciculus white matter integrity (FA)	-32.75158208	0.044521963
DTI posterior corpus callosum white matter integrity (FA)	-31.36531586	0.0459431
DTI right inferior fronto-occipital fasciculus white matter integrity (FA)	-46.36084129	0.047172523
Resting state right amygdala - right subcallosal cortex	2.938996577	0.049110556
rs9675994 biomarker	1.059860946	0.050472715
Resting state right amygdala - left Heschl's gyrus	4.030838071	0.050983394
DTI left superior longitudinal fasciculus white matter integrity (FA)	-33.69284771	0.055707869
rs8092433 biomarker	1.341891667	0.056891673
Resting state right amygdala - right precentral gyrus	3.274875153	0.059176308
rs1629089 biomarker	-1.244919391	0.061284572
rs4890888 biomarker	1.275922167	0.066888504
Resting state left amygdala - left subcallosal cortex	3.287378249	0.071091156
rs10221416 biomarker	-1.341547504	0.071757763
Resting state right amygdala - left precentral gyrus	3.059812373	0.07640019
ERP Average Latency Ratio - OZ	-0.76408982	0.077552746
Resting state right amygdala - left planum temporale	3.103178681	0.07960113
rs16965628 biomarker	1.285489301	0.08067999
rs9979500 biomarker	-1.271878886	0.083906644
Resting state right amygdala - left postcentral gyrus	2.65299295	0.09001391
DTI right inferior fronto-occipital fasciculus white matter integrity (FA)	-34.47621509	0.093118753
DTI right inferior longitudinal fasciculus white matter volume	-0.000315719	0.095959938
DTI left inferior longitudinal fasciculus white matter volume	-0.000346706	0.097055856
rs2282574 biomarker	-0.998681054	0.101870037
Resting mean SCL	-0.311805452	0.10303958
rs12273539 biomarker	-1.064940027	0.104406601
rs16917234 biomarker	-1.064940027	0.104406601
Resting state right amygdala - right supplementary motor cortex	2.842306697	0.106797333
rs470478 biomarker	-2.367123614	0.109072396
rs1789076 biomarker	2.367123614	0.109072396
DTI right uncinate fasciculus white matter integrity (FA)	-26.48894197	0.109289789
rs3794834 biomarker	0.914138483	0.109743119
DTI right optic radiation white matter volume	-0.001088728	0.110673232
rs1026520 biomarker	-0.872594113	0.111659568
DTI right inferior longitudinal fasciculus white matter volume	-0.000379376	0.115855258
rs9964845 biomarker	1.019288	0.115939989
rs8185002 biomarker	1.704023836	0.116337935
DTI tapetum white matter integrity (FA)	-21.18022861	0.118036648
rs40184 biomarker	-0.966551612	0.119241285
DTI left optic radiation white matter volume	-0.001631125	0.122493042
DTI right cingulum white matter integrity (FA)	-30.41818634	0.124148772
rs2839350 biomarker	1.661710233	0.128038559
NPY baseline assay	0.026935972	0.128294092
Resting state right amygdala - left parietal operculum cortex	2.478271555	0.129042055
DTI middle cerebellar peduncle white matter volume	-0.000448081	0.134419753
Resting state right amygdala - right postcentral gyrus	2.543224308	0.137747162
DTI left optic radiation white matter integrity (FA)	-20.57454	0.13813035
Resting state right amygdala - right planum temporale	2.717433586	0.144717558
rs8099667 biomarker	-1.369487243	0.145147178
rs2282566 biomarker	0.986877467	0.14803593
ERP Average Latency Ratio - C4	1.399460125	0.148636917

**Table 2:** Excluded variables based on variance inflation factor (VIF) of multicollinearity and the high degree of missingness Baseline measures with VIF > 10 represent a high level of multicollinearity and were excluded before performing multiple regression analysis. * indicates those that were excluded based on missing data.

Variables with VIF > 10
rs8092433 biomarker
Resting state right amygdala - left precentral gyrus
DTI posterior corpus callosum white matter integrity (FA)
DTI right posterior thalamus radiation white matter integrity (FA)
DTI left inferior longitudinal fasciculus white matter integrity (FA)
DTI right superior longitudinal fasciculus white matter integrity (FA)
DTI tapetum white matter integrity (FA)
DTI left superior longitudinal fasciculus white matter integrity (FA)
Resting state right amygdala - right subcallosal cortex
DTI right inferior fronto-occipital fasciculus white matter integrity (FA)
Resting state right amygdala - left postcentral gyrus
Resting state right amygdala - right precentral gyrus
Resting state right amygdala - left parietal operculum cortex
DTI left optic radiation white matter integrity (FA)
DTI right inferior longitudinal fasciculus white matter integrity (FA)
Resting state right amygdala - left planum temporale
Resting state right amygdala - right postcentral gyrus
DTI left inferior longitudinal fasciculus white matter volume
Resting state left amygdala - left subcallosal cortex
ERP Average Latency Ratio - PZ
ERP Average Latency Ratio - OZ
Ethnicity*

**Table 3:** Multivariate analysis using ensemble tree models - Random forests approach results The random forest procedure was performed using 1000 trees on the same 49 variables obtained after univariate and multicollinearity analysis. The top important variables strongly associated with the outcome are listed based on positive variable importance (VIMP).

Variable	VIMP
Resting state right amygdala - right supplementary motor cortex	9.322006
rs3794834 biomarker	4.557822
Resting state right amygdala - left Heschl's gyrus	4.338526
NPY baseline assay	3.645515
rs1629089 biomarker	3.325563
rs8185002 biomarker	2.743513
Resting state right amygdala - left subcallosal cortex	2.585636
rs2008323 biomarker	2.421426
DTI right optic radiation white matter (FA)	2.110193
rs8099667 biomarker	1.925468
rs9903602 biomarker	1.740449
rs2282574 biomarker	1.692296
rs2839350 biomarker	1.580902
rs642506 biomarker	1.46731
rs40184 biomarker	0.983404
Resting mean SCL	0.753201
DTI right uncinate fasciculus white matter integrity (FA)	0.690579
rs12273539 biomarker	0.506212
DTI right superior longitudinal fasciculus white matter volume	0.071362
